# Using Mitochondrial Trifunctional Protein Deficiency to Understand Maternal Health

**DOI:** 10.33696/signaling.1.018

**Published:** 2020

**Authors:** Jason W. Miklas, Hannele Ruohola-Baker

**Affiliations:** 1 Institute for Stem Cell and Regenerative Medicine, University of Washington, School of Medicine, Seattle, WA 98109, USA; 2 Department of Bioengineering, University of Washington, Seattle, WA 98195, USA; 3 Department of Biochemistry, University of Washington, School of Medicine, Seattle, WA 98195, USA

## Abstract

Fatty acid oxidation disorders unfortunately can result in the sudden unexplained death of infants. Mitochondrial trifunctional protein (MTP) deficiency is one such disease where long-chain fatty acids cannot be fully oxidized through beta-oxidation which, can lead to cardiac arrythmias in an infant. Furthermore, mothers who are carrying an MTP deficient fetus have a prevalence for pregnancy complications, especially AFLP, acute fatty liver of pregnancy and HELLP syndrome. To better understand the etiology of the potential pro-arrhythmic state the MTP deficient infants may enter, we developed an *in vitro* model of MTP deficiency in cardiomyocytes to elucidate the underpinning molecular mechanism of this disease. Using CRISPR/Cas9, we developed MTP deficient mutant and knockout pluripotent stem cell lines. Furthermore, we generated patient derived induced pluripotent stem cell lines harboring a so-called founder mutation, the most commonly identified alteration in MTP in the population. Upon differentiating these mutant stem cells into cardiomyocytes and then challenging with fatty acids, we observed pro-arrhythmic behavior, depressed mitochondrial energetics, and elevated hydroxylated long-chain fatty acids, all perhaps expected phenotypes due to MTP deficiency. However, unexpectedly, we also identified an inability of these disease cardiomyocytes to generate mature cardiolipin. Cardiolipin is a key pillar of the powerhouse of life, mitochondria. For the first time this disease-in-a-dish model revealed the key culprit for the dramatic MTP mutant mitochondrial defects and identified potentially a second role for the enzyme HADHA in MTP. HADHA is required for the biosynthesis of functional cardiolipin and therefore healthy mitochondria. However, in the disease, defective cardiolipin results in mitochondrial abnormalities and cardiac arrythmias in infants. These studies reveal an important target for sudden infant death syndrome therapy. With this foundational work on an *in vitro* model of MTP deficiency and potential avenues for therapy, the next important task is to extend this model to address fetal-maternal interactions towards better governing maternal health.

Mitochondrial trifunctional protein (MTP) deficiency is a disease that manifests in the cardiac system after birth. This is thought to occur due to the change in newborn diet at birth where the infant begins consumption of a mother’s breast milk, which is full of many essential and important molecules, including antibodies. Unfortunately for MTP deficient infants, breast milk is also high in fats, especially in long-chain fatty acids that MTP patients can not break down. This process then precipitates in disease pathology.

MTP deficiency is caused by mutations in either hydroxyacyl-CoA dehydrogenase/3-ketoacyl-CoA thiolase /enoyl-CoA hydratase subunit A (HADHA) or subunit B (HADHB). The result is a severe limitation in long-chain fatty acid oxidation (FAO) and is considered one of the more severe FAO disorders without pharmacological treatment [1]. Mitochondrial FAO disorders are recessively inherited, and defects in FAO are estimated to affect ~1 in 10,000 newborns [2]. This results in around 400 infants per year born in the USA with this condition that is potentially lethal and has no cure. In particular regions of Poland the frequency is over ten-fold higher [3]. Unfortunately, a mother carrying a MTP deficient fetus has a 30% chance of exhibiting pregnancy complications [4]. In particular, if the mother has a mutation in one copy of *HADHA* and the fetus has two copies of the *HADHA* mutation, the mother is more likely to have acute fatty liver of pregnancy (AFLP) and HELLP syndrome (hemolysis, elevated liver enzymes and low platelet count). Yet, little is known about how the mother develops AFLP and/or HELLP syndrome. MTP deficiency and human stem cell derived cell types provide an opportunity to better understand human cell-to-cell communication in human physiology and disease pathology [5].

MTP deficiency results in sudden unexplained infant death, Reye-like syndrome, cardiomyopathy and/or skeletal myopathy [1,6,7]. A major phenotype of MTP-deficient newborns is sudden infant death syndrome (SIDS), which manifests after birth once the child begins nursing on lipid-rich breast milk. Defects in FAO have a role in promoting a pro-arrhythmic cardiac environment, however, the exact mechanism of action was not understood, and there are no current therapies [8,9]. We (Miklas et al.) recently used human induced pluripotent stem cells from a HADHA patient and HADHA CRISPR edited iPSCs to model human MTP deficient cardiomyocytes, heart cells, to better understand the cardiac pathology [5]. Two key findings were made. The first was that HADHA mutant cardiomyocytes, when challenged with fatty acids, had defective calcium dynamics and repolarization kinetics which resulted in a pro-arrhythmic state. Second, defective HADHA led to abnormal cardiolipin remodeling resulting in the inability to produce and possibly maintain the acyl-chain composition of mature cardiolipin [10].

Cardiolipins are a critical component of the mitochondrial inner membrane. Cardiolipin is an atypical phospholipid composed of four (instead of two) acyl-chains that are connected with a glycerol moiety. This atypical structure of cardiolipin results in a conical shape that is thought to be critical for inner mitochondrial membrane structure and function [11]. In particular, cardiolipin has been shown to function in organizing the electron transport chain (ETC) higher order structure, important for ETC activity, and acts as a proton trap on the outer leaflet of the inner mitochondrial membrane [12]. Hence, the reduction of the mature form of cardiolipin results in mitochondrial abnormalities such as proton gradient loss, ETC depression resulting in depressed ATP production and abnormal mitochondrial architecture [13].

Pathological remodeling of cardiolipin has been implicated previously in the mitochondrial dysfunction observed in diabetes, heart failure, neurodegeneration, and aging [13,14]. However, the pattern and composition of abnormal cardiolipin species in the case of the HADHA mutant and knockout cardiomyocytes were more specific than seen previously in heart failure or diabetes, suggesting that HADHA may be directly involved in cardiolipin processing. Interestingly, previous studies using HeLa cells have suggested HADHA exhibits acyl-CoA transferase activity upon monolyso-cardiolipin for its remodeling into cardiolipin [15]. As such, these data suggest that defects in HADHA directly cause impaired cardiolipin remodeling resulting in the inability to produce and possibly maintain the acyl-chain composition of mature cardiolipin [10]. However, the exact contribution of this acyltransferase to physiological cardiolipin remodeling has been unclear [15]. Miklas et al. report that, FA challenged human HADHA mutant and knockout cardiomyocytes have reduced mature tetra[18:2]-cardiolipin and compromised mitochondrial activity (Figure 1). This is similar to previously seen findings in tafazzin mutants causing Barth’s syndrome [16], an X-linked cardiac and skeletal mitochondrial myopathy [16,17]. These data, for the first time, establish the exact contribution of HADHA acyltransferase to physiological cardiolipin remodeling in human cardiomyocytes and open the door for future treatments and therapies. In particular, cardiolipin has previously been considered as a druggable target and the existing and future molecules affecting and stabilizing cardiolipin have a strong potential to become therapeutic agents for SIDS.

## Model maternal pregnancy complications using MTP deficient stem cell derived placental cells

Now that the foundational cell culture framework has been generated to study MTP deficiency *in vitro* in cardiomyocytes [5], it is important in the future to extend this *in vitro* model to better understand AFLP and HELLP syndrome that occur during pregnancy [18]. Using recent advances in stem cell biology, it is possible to stably culture trophoblast stem cells, the cells that form the placenta, either directly from an embryo [19] or by reprogramming embryonic stem cells [20]. It has been shown that the placenta has a large amount of fatty acid oxidation enzymes present, including mitochondrial tri-functional protein [21]. It is hypothesized that placental tissue may take up free fatty acids from the mother’s blood and incorrectly process those fatty acids in a similar manner as shown in our work and patients. As a result, an intermediate fatty acid, such as hydroxylated long-chain fatty acids, may accumulate and be exported back into the mother’s blood from the placental tissue. However, the specific lipid substrates the placental tissue uses for fatty acid oxidation, what fatty acid substrates might be transported to the fetus, and whether or not processed fatty acids can be exported back into the mother’s blood remain unknown. By using trophoblast stem cells to generate the working cells found in the placenta, such as the villous cytotrophoblast and/or extravillous trophoblast [22], one could use global lipidomics techniques to assess fatty acid content of these cells and their supernatant in the wild type and HADHA knockout state. By better understanding the fundamental fatty acid metabolism underlying placental biology, during health and disease, has the potential to better understand the fundamental way in which development occurs and the role the placenta has in being a gatekeeper of certain metabolic substrates for the fetus.

**Figure 2:**
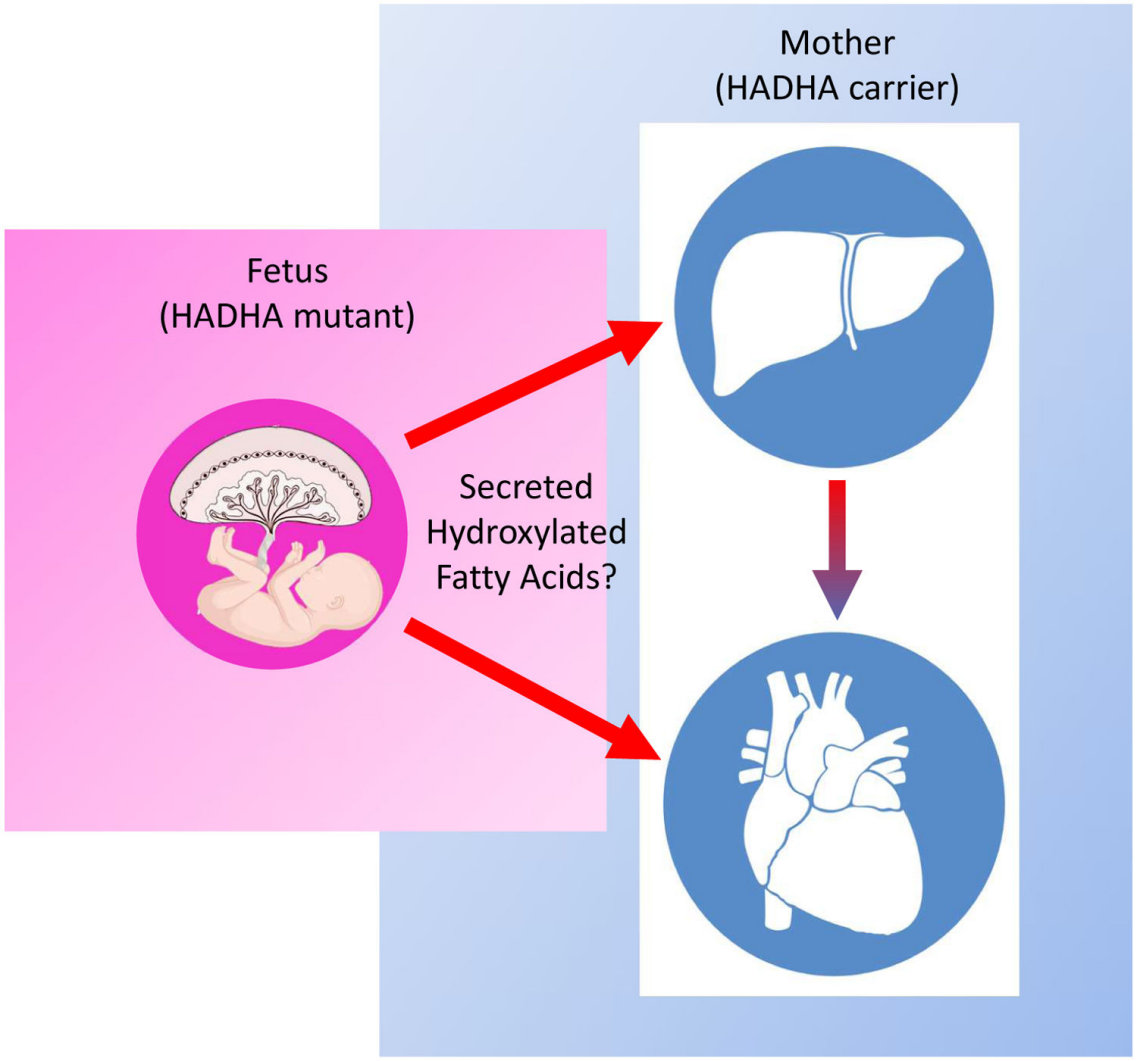
Modeling the effects of fatty acids secreted from HADHA mutant fetal placental cells to mother’s liver and heart. Potentially, the placental tissue takes up long-chain fatty acids from the mother’s blood. However, due to the inability to breakdown the long-chain fatty acid via β-oxidation, the hydroxylated fatty acid intermediate builds up and is exported back into the mother’s blood stream. Consequently, these hydroxylated fatty acids and other mis-processed long-chain fatty acids may be taken up by the mother’s heart and liver resulting in various complications during pregnancy. This figure was made in part with BioRender.

Finally, leveraging human-on-a-chip co-culture *in vitro* platforms [23], organ-organ lipid communication can be modeled to understand how, placental tissue lacking the *HADHA* gene may impact the health of a heterozygous mother’s liver to result in AFLP and HELLP syndrome (Figure 2). In doing so, ideally a causal lipid or set of lipids may be identified that result in exacerbating the mother’s liver’s limited ability to process long-chain fatty-acids resulting in these complications. Furthermore, using such a platform, novel drug, protein or gene interventions can be tested to identify means of rescuing aberrant lipid molecule signaling. These findings could have generalizable uses in the wider array of AFLP and HEELP syndromes complications that unfortunately arise during so many pregnancies worldwide.

## Figures and Tables

**Figure 1:**
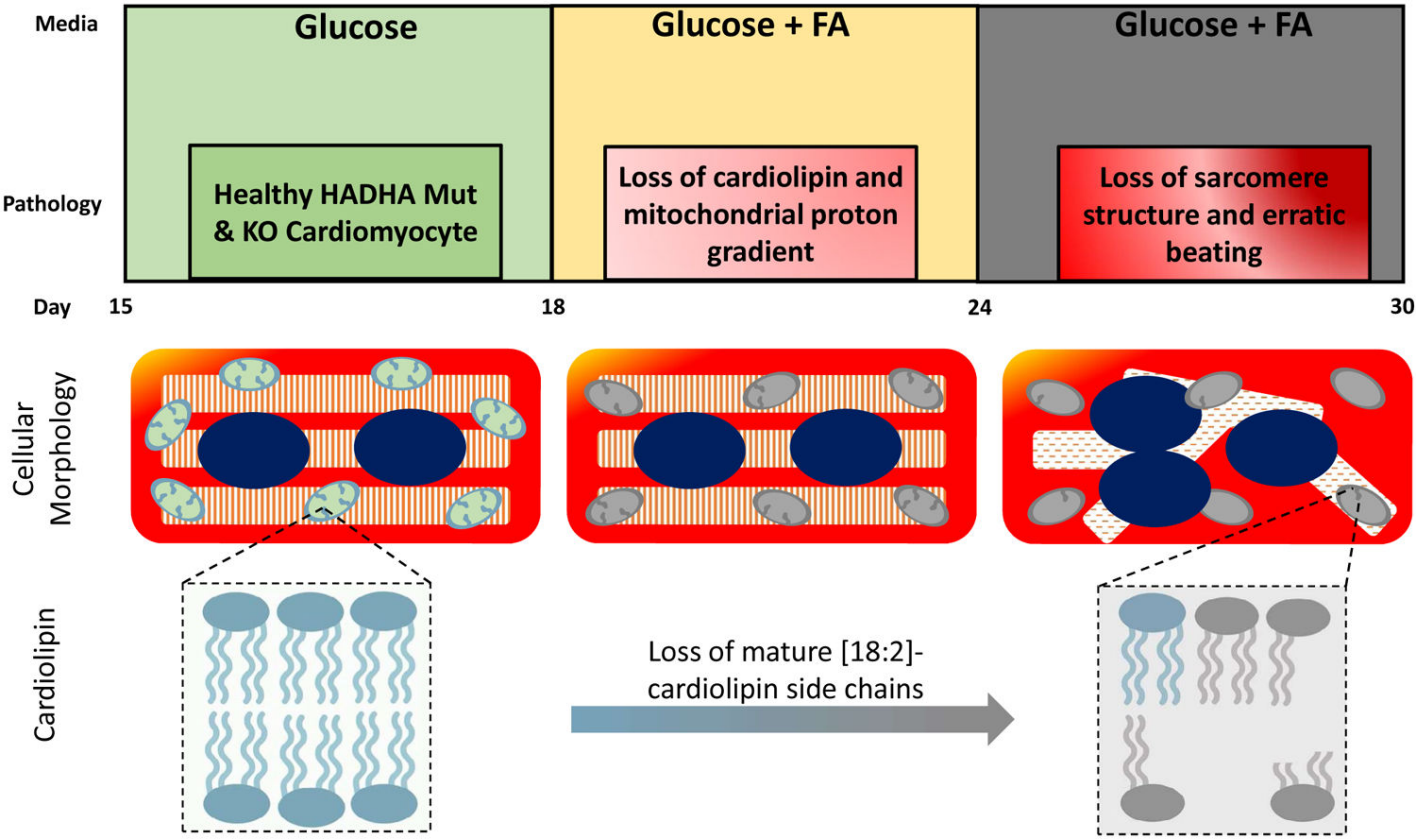
Summary of HADHA mutant cardiomyocyte disease phenotype progression. Differentiating cardiomyocytes from stem cells takes about 12 to 15 days. Directly after differentiation cardiomyocytes are cultured in a glucose rich media. In this media, HADHA mutant and knockout (KO) cells show normal cardiomyocyte physiology. 18 days after differentiation, cells are transferred into media containing glucose and a cocktail of long-chain fatty acids. Soon after fatty acid exposure, HADHA mutant and knockout cardiomyocytes show loss of mature cardiolipin, with four linoleic-acid side chains ([18:2]_4_) and a reduction in mitochondrial proton gradient. Further culturing with long-chain fatty acids results in cardiomyocytes losing sarcomere structure and begin to display erratic beating suggesting a pro-arrhythmic state. This figure was made in part with BioRender.
